# Effect of *In Vitro* Exposure of Corticosteroid Drugs, Conventionally Used in AMD Treatment, on Mesenchymal Stem Cells

**DOI:** 10.1155/2012/946090

**Published:** 2012-05-23

**Authors:** Raffaele Nuzzi, Monica Gunetti, Deborah Rustichelli, Barbara Roagna, Francesca Fronticelli Bardelli, Franca Fagioli, Ivana Ferrero

**Affiliations:** ^1^Department of Clinical Pathophysiology, Ophthalmology Section, University of Turin, 10126 Turin, Italy; ^2^Pediatric Onco-Hematology, Stem Cell Transplantation and Cellular Therapy Division, Regina Margherita Children's Hospital, 10126 Turin, Italy; ^3^Department of Pediatrics, University of Turin, Piazza Polonia 94, 10126 Turin, Italy

## Abstract

Age-related macular degeneration (AMD) is a leading cause of legal blindness in individuals over 60 years of age, characterized by the dysfunction of retinal pigmented epithelium cells, specifically in the macular area. Despite several treatment options, AMD therapy remains difficult, especially for exudative AMD. Multipotent mesenchymal stem cells (MSCs), with great plasticity and immunomodulant properties, are a promising cell source for cellular therapy and tissue engineering. We evaluated the effects of steroid drugs, often used to treat AMD, in association with MSCs, in view of a possible application together to treat AMD. Morphology, viability, growth kinetics, and immunophenotype were evaluated on healthy donors' MSCs, treated with triamcinolone acetonide, alcohol-free triamcinolone acetonide, micronized intravitreal triamcinolone and dexamethasone at different concentrations, and in a human retinal pigment epithelial cell line supernatant (ARPE-19). The morphological analysis of MSCs in their standard medium showed a negative correlation with drug concentrations, due to the numerous crystals. Dexamethasone was the least toxic corticosteroid used in this study. ARPE-19 seemed to help cells preserve the typical MSC morphology. In conclusion, this *in vitro* study demonstrated that high doses of corticosteroid drugs have a negative effect on MSCs, reduced in the presence of a conditioned media.

## 1. Introduction

Age-related macular degeneration (AMD) is a leading cause of legal blindness in developed countries in individuals over 60 years of age [1]. It is characterized by the dysfunction of Retinal Pigmented Epithelium (RPE) cells, specifically in the macular area. As a result, debris accumulates within these cells and also form drusen, discrete deposits of proteins and lipids between Bruch's membrane and the RPE, [2]. Secondly, the photoreceptor cells degenerate, due to the loss of RPE function and nutritional support. Two types of AMD are known. The dry or non-exudative form accounts for about 90% of all cases and it is characterized by a gradual and progressive loss of visual function up to the development of geographic atrophy. The wet or exudative form is associated with the development of choroidal neovascularization (CNV) that leads to a sudden and dramatic central visual activity loss. 

There are few treatment options for the dry form and mainly consist of a high-dose of an oral combination of the antioxidants ascorbic acid (vitamin C), tocopherol (vitamin E), and beta-carotene, in addition to copper and zinc. Therefore, therapeutic approaches for AMD are almost exclusively focused upon the exudative form and are only of limited benefit to most patients. Despite the recent advent of several treatment options, AMD treatment remains difficult, especially for exudative AMD.

Photodynamic therapy utilizes the production of a selective cytotoxic effect that causes nonthermal photo-thrombosis on pathological vessels [3, 4]. Corticosteroids have a number of positive effects in the treatment of neovascular lesions, having a strong anti-inflammatory, antiproliferative, and antiangiogenetic action [5] and may also be useful to limit some adverse events caused by photodynamic therapy. In ophthalmology, despite their treatment benefits, side effects, including ocular toxicity, have been observed especially when intraocular delivery is used. 

Conventional steroid drugs such as Triamcinolone acetonide (9a-fluoro-16a-hydroxyprednisolone, TA), a synthetic crystalline corticosteroid with potent anti-inflammatory properties, Intra Vitreal Triamcinolone (IVT), a micronized formulation approved for surgical use, and Ozurdex (Allergan, Inc. Irvine, CA, USA), a pharmaceutical formulation with 0.7 mg of dexamethasone, are used in ophthalmological therapy by periorbital injection, and today represent an adjuvant treatment in exudative AMD and proliferative vitreoretinopathy [6–9].

In addition to the standard treatments for AMD, new emerging therapies such as stem cell therapy are being developed. Stem cell transplantation is a promising approach for degenerative diseases such as Retinitis Pigmentosa, Stargardt disease, AMD, and other retinal degeneration that are still incurable in most cases.

Multipotent mesenchymal stem cells (MSCs) are a promising cell source for cellular therapy and tissue engineering because of their great plasticity [10, 11] and ability to provide the host tissue with growth factors or modulate the host immune system [12]. MSCs can easily be isolated from bone marrow thanks to their capacity to adhere and proliferate and expand in culture while maintaining their immunophenotypical characteristics and functions as multipotent cells [13]. They can also produce multiple cytokines, growth factor, and adhesion molecules, all important factors which influence the hematopoietic microenvironment.

MSCs are also known to exert immunosuppressive effects, and to secrete neurotrophic factors [14], and have anti-inflammatory and antiproliferative effects on microglial cells and astrocytes, resulting in the induction of a neuroprotective microenvironment [15]. They can be safely cultured *in vitro* with no risk of malignant transformation [16].

*In vitro* and *in vivo* studies showed that MSCs can differentiate into retinal neurons [17], and that the sub-retinal transplantation of MSCs delays retinal degeneration and preserves retinal function [18]. Inoue demonstrated that MSC transplantation into the sub-retinal space of RCS rats (a retinal degeneration model) delays retinal degeneration and preserves retinal function in the RCS rats, suggesting that MSCs are a useful cell source for cell-replacement therapy for some forms of retinal degeneration [19]. Furthermore, umbilical-derived mesenchymal stem cells proved effective in sustaining visual function for several months after injection into the sub-retinal space of RCS rats [20]. 

Given the lack of treatments for dry AMD, and the time-consuming and expensive nature of treatment for wet AMD, AMD is a perfect candidate for the application of stem cell therapy. Previous studies for other nonocular diseases have tested the use of stem cells in combination with corticosteroids, pointing to positive effects on cell adhesion, proliferation, and viability. Corticosteroids can induce cell fate and differentiation cascades, with strong evidence in both clinical and basic science experiences. These drugs may therefore stimulate the proliferation and differentiation of MSCs according to the complex environmental conditions [21], that play an essential role in inducing cell fate and differentiation cascades of stem cells in culture. Exploring the impact of these drugs on MSCs holds promise in revealing important details of stem cell biology and in finding new fields of possible therapeutic applications.

In order to evaluate the possibility to treat AMD by MSCs associated with conventional steroid, in this study, we evaluated the effects of steroid drugs, very common drugs often used to treat AMD, in association with MSCs. We tested morphology, viability, growth kinetics, and immunophenotype.

## 2. Material and Methods

### 2.1. Isolation and Expansion of MSCs

MSCs were isolated from bone marrow (BM) collected from healthy donors bone marrow (BM) harvested from the iliac crest of adult or pediatric Caucasian donors who underwent bone marrow collection for a related patient after written informed consent. Whole BM was layered on Percoll (Sigma Aldrich, St. Louis, MO, USA) gradient (density: 1.073 g/mL) and centrifuged at 1,100 g for 30 minutes. The cells in the interphase were washed twice with PBS1X (200 g for 10 minutes) and seeded at a density of 800,000/cm^2^ in MSC Medium (Lonza, Basel, Switzerland) at 10% of Fetal Bovine Serum (FBS, Lonza) in 75 or 150 cm^2^ T-flasks (Falcon) and maintained at 37°C with an atmosphere of 5% CO_2_. After 3 days, the nonadherent cells were removed and the cultures re-feeded every 3-4 days. At confluence, after about 15 days, the adhered monolayer was detached with trypsin/EDTA (Lonza) for 5 minutes at 37°C, and the trypsin action was blocked with trypsin neutralizing solution (Lonza) for 5 minutes at 37°C. The cells were then seeded at a density of 8,000/cm^2^ and detached every 7 days for 2-3 passages in order to expand the isolated cells.

MSCs were characterized according to the International Society for Cellular Therapy (ISCT) Guide Lines [13]. To test MSC differentiative potential, MSCs were cultured in osteogenic, adipogenic, and chondrogenic media and analyzed as previously reported [22]. 

### 2.2. Drugs

Triamcinolone acetonide (TA, Kenacort, Bristol-Meyers Squibb), alcohol-free Triamcinolone acetonide (AF-TA, obtained by microfiltration of TA and dilution in physiological solution), micronized intravitreal triamcinolone (IVT, Sooft Italia), Dexamethasone 21-fosfato disodico (Dex, Decadron phosphate, Merck Sharp & Dohme) were tested *in vitro* at different concentrations (0.01 mg/mL, 0.1 mg/mL, and 1.0 mg/mL).

### 2.3. Drug Evaluation on MSCs

MSCs were seeded with the drugs at different concentrations after 4 culture passages. After 24, 72 hours, and 5 days, the morphology, viability, and immunophenotype were evaluated by cytofluorimetric analysis. 

### 2.4. Drug Evaluation on MSCs in the Presence of Retina Cells

The human retinal pigment epithelial cell line ARPE-19 [23] (ATCC, LGC Standards; Milan Italy) was maintained in Dulbecco's modified Eagle's medium in Ham's F12 (DMEM/F12). The cells were detached every 5–7 days with trypsin/EDTA (Lonza) for 5 minutes at 37°C, and the supernatant (SN) was collected, filtered and stocked at −20°C.

MSCs at 2–4 passages were cultured with 50% or 100% ARPE-19 SN and maintained in culture changing medium every 3-4 days.

We evaluated the toxicity of steroid drugs at different concentrations on MSCs with or without the supernatant of retinal cell culture ARPE-19 at 50% or 100% concentrations. After 24, 72 hours, and 5 days, the morphology, viability, and immunophenotype were evaluated by cytofluorimetric analysis.

### 2.5. MSC Analysis

The immunophenotype analysis on MSCs was performed by flow cytometry on 200,000 cells, which were incubated for 20 minutes at 4°C with fluorescein-(FITC-) or phycoerytrin-(PE-) conjugated monoclonal antibodies anti-CD45, CD14 (Becton Dickinson, San Jose, CA, USA), CD90, CD29, CD73, and CD105 (Caltag Laboratories, Burlingame, CA, USA), as ISCT guidelines suggest [13]. After 1 wash in PBS 1X, the cells were resuspended in 200 *μ*L of PBS 1X and analysed on Epics-XL cytometer (Beckman Coulter, CA, USA). The positive cell percentage was calculated using cells stained with Ig FITC/PE as a negative control.

### 2.6. Immunofluorescence

MSCs cultured in presence of ARPE-19 SN were evaluated by immunofluorescence for retinal markers RPE65, Opsin, and PKC, after 7 and 14 days.

The cells were fixed and permeabilized with acetone-methanol (1 : 1) for 20 minutes at −20°C. The fixed cells were washed with PBS 1X (Cambrex, Belgium), and nonspecific binding was blocked with 0.1% human albumin (HSA) in PBS for 1 hour at room temperature (RT). The cells were incubated with the primary antibody anti RPE65 (mouse), Opsine (rabbit), and PKC (rabbit) and then with CY3 anti-rabbit (Immunological Sciences, Rome, Italy; 1 : 1000), or AlexaFluor 488-coupled anti-mouse (1 : 500, Southern Biotechnology, Birmingham, AL, USA). Positive cells were counted and compared to total cell counts labelled with 4′,6-diamidino-2-phenylindole (DAPI, Molecular Probe). The cells were examined under epifluorescence microscopy (Axiovert 200, Carl Zeiss, AG, Germany) and analysed by AxioVision Rel 4.2 (Carl Zeiss, AG, Germany). Magnification 20× e 40×.

## 3. Results

**Figure 1:**
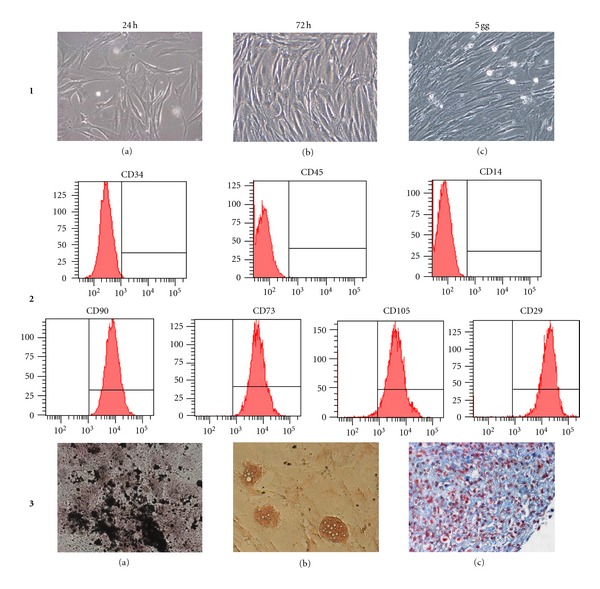
Characterization of MSCs according to ISCT guidelines. Panel 1: spindle-shaped cells typical of MSCs after 24 hrs, 72 hrs, and 5 days from seeding. Panel 2: immunophenotypic analysis of MSCs showing the negativity of CD34, CD45 and CD14 expression and the positivity of CD90, CD73, CD105, and CD29. Panel 3: differentiative potential of a representative MSC: presence of calcium ossalates observed in Van Kossa staining (a) after osteogenic induction; presence of lipid intracytoplasmic vacuoles stained with Oil Red O (b) after adipogenic induction and presence of hjaluronic acid by Alcian Blue staining (c) after chondrogenic induction. Original magnification 40× (a, c) and 20× (b).

MSCs were isolated from BM and characterized according to the ISCT Guide Lines (Figure 1). In order to evaluate the possibility to treat AMD by MSCs associated with conventional steroid, we tested the morphology, viability, growth kinetics, and immunophenotype which were then evaluated on MSCs treated with the different drugs, (TA, AF-TA, IVT, and Dex).

### 3.1. Morphology

**Figure 2:**
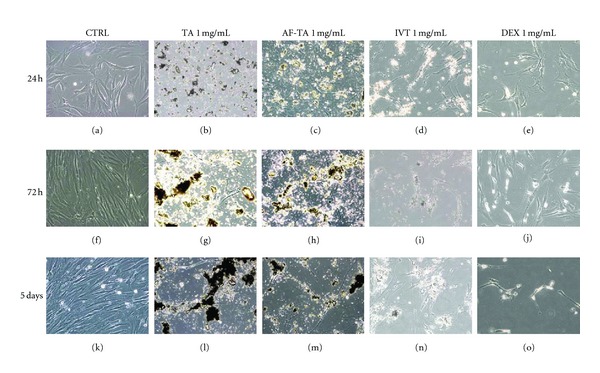
Morphological analysis of MSCs in the presence of drugs at 1 mg/mL concentration. Basal MSCs at 24 hrs, 72 hrs, and 5 days (a, f, k); TA at 24 hs, 72 hs, and 5 days (b, g, l); AF-TA at 24 hs, 72 hrs, and 5 days (c, h, m); IVT at 24 hrs, 72 hrs, and 5 days (d, i, n); DEX at 24 hrs, 72 hrs, and 5 days (e, j, o). Original magnification 20×.

Phase contrast microscopy showed a clumping of TA crystals. The morphological analysis of MSCs in MSC medium showed a high level of toxicity in correlation with the drug concentration, because of the presence of numerous crystals, especially when the cells were treated with 1 mg/mL AF-TA. The same phenomenon was evident with TA in alcohol solution after 72 hours and 5 days, in a more marked way with 1 mg/mL formulations (both with and without alcohol). With 1 mg/mL IVT and, above all, with Dex, the morphology was better preserved and fewer precipitates were present, compared to TA (Figure 2).

**Figure 3:**
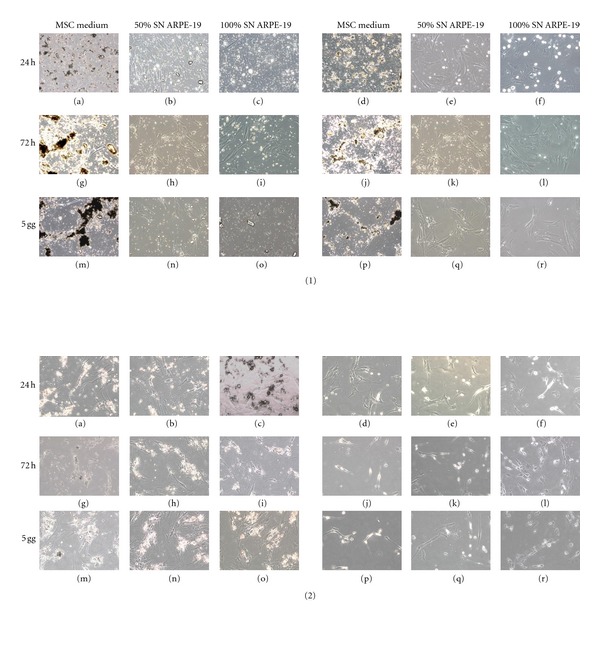
Morphological analysis of MSCs in the presence of drugs at 1 mg/mL concentration and ARPE-19 SN. Panel 1: MSCs with TA 1 mg/mL at 24 hrs (a, b, c), 72 hrs (g, h, i), and 5 days (m, n, o); AF-TA 1 mg/mL at 24 hrs (d, e, f), 72 hrs (j, k, l), and 5 days (p, q, r); Panel 2: IVT 1 mg/mL at 24 hrs (a, b, c), 72 (g, h, i), and 5 days (m, n, o); DEX 1 mg/mL at 24 hrs (d, e, f), 72 hrs (j, k, l), and 5 days (p, q, r). Original magnification 20×.

When MSCs were maintained with drugs and ARPE-19 SN, the morphology analysis revealed a smaller presence of drug crystals (Figure 3).

### 3.2. Viability

TA showed no toxic effects at 0.01 mg/mL: viability was 94.20%, 97.10%, and 98.50% after 24, 72 hours, and 5 days, respectively. TA 0.1 mg/mL involves a fall of viability at 24 hours (65.50%) and a restoration of cultures after 72 hours (91%) and 5 days (93%).

AF-TA involves a slight fall of viability at 0.01 mg/mL, while at 0.1 mg/mL viability falls to 76.50% and 77% after 24 and 72 hours, respectively, with a resumption after 5 days.

IVT was toxic only after 5 days of culture at 0.1 and 1 mg/mL.

**Figure 4:**
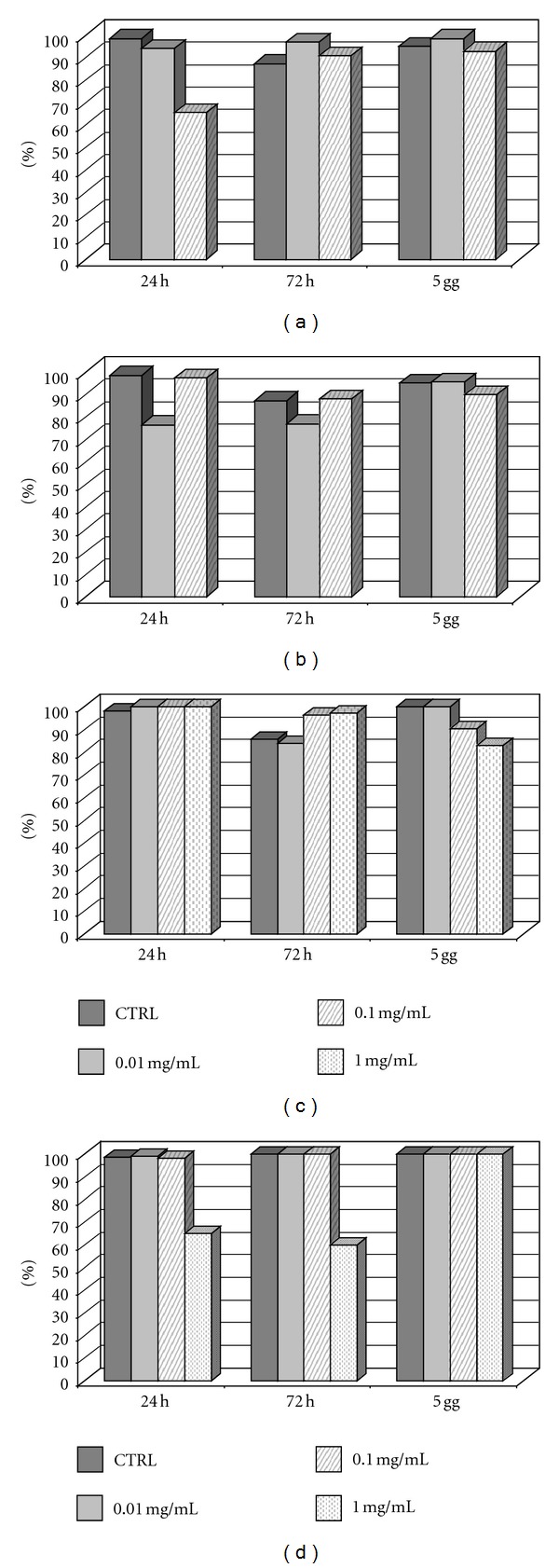
Viability conditions at different concentrations of triamcinolone acetonide (a), alcohol-free triamcinolone (b), IVT (c), and Dexamethasone (d).

Dexamethasone was very toxic at 1 mg/mL after 24 and 72 hours, while at 0.01 mg/mL and 0.1 mg/mL the cells remained viable at each time of analysis. All these data are showed in Figure 4.

### 3.3. Growth Kinetics

The cellular expansion growth rate of MSCs was evaluated by cell count in a Burker chamber at each passage and expressed in terms of fold increase.

**Figure 5:**
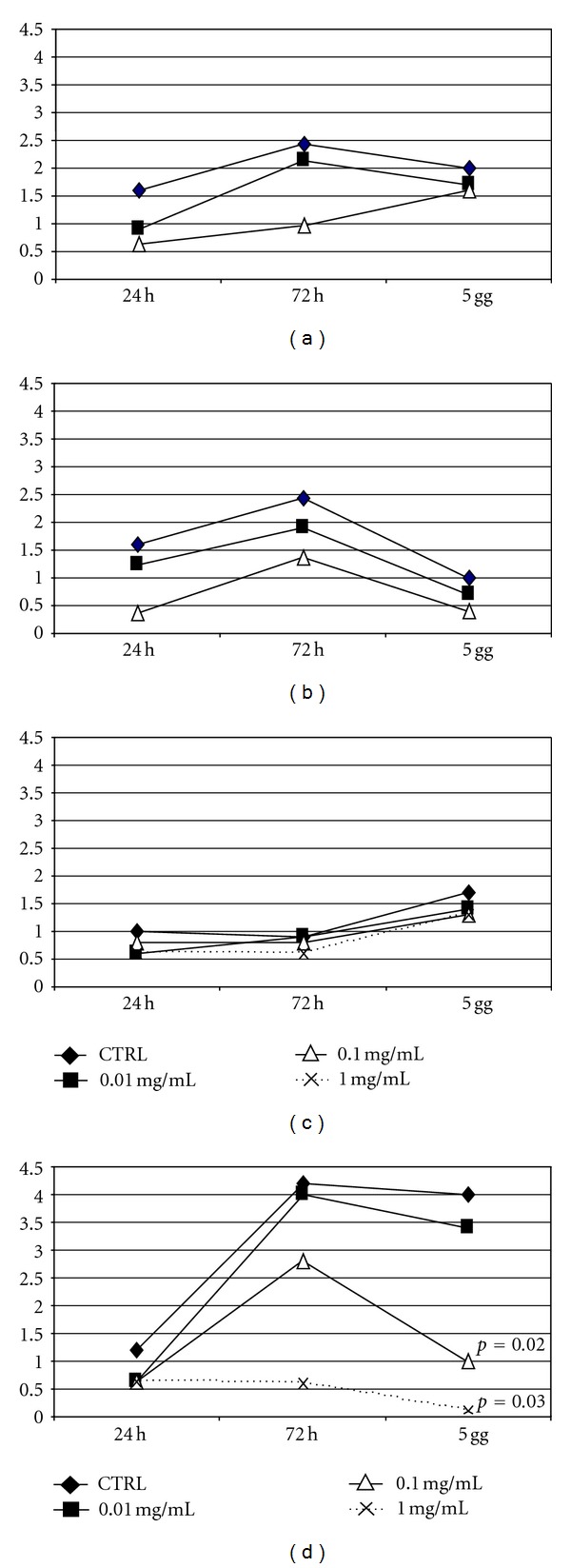
Growth rate at different concentrations of triamcinolone acetonide (a), alcohol-free triamcinolone (b), IVT (c), and Dexamethasone (d). After 5 days' culture the cellular growth decreases at 0.1 and 1 mg/mL dexamethasone (*P* = 0.02, and *P* = 0.03 resp.).

All drugs had a negative effect on cellular growth. Dexamethasone inhibits MSC growth rate especially at 0.1 and 1 mg/mL after 5 days' culture (*P* < 0.05) (Figure 5).

### 3.4. Immunophenotype

During the experiments, MSCs were negative for the haematopoietic antigen (CD34, CD45 and CD14), and expressed high percentages of CD90, CD105, CD29, and CD73 (data not shown). Immunophenotype analysis showed a negative effect, after 24 hours of TA and AF-TA on mesenchymal antigen expression, mostly at 0.1 mg/mL. However, after 72 hours and 5 days of culture there is a restoration of antigen expression, with slight decreases on the fifth day. IVT at 1 mg/mL exposition induced a decreased expression of MSC antigens after 24 hours (*P* < 0.05), and after 72 hours and 5 days. Dexamethasone did not show a negative effect on antigens expression. All drugs at 1 mg/mL, however, induced an altered morphology of cells that did not permit the cytofluorimetric analysis, even after 24 hours.

### 3.5. Immunofluorescence

**Figure 6:**
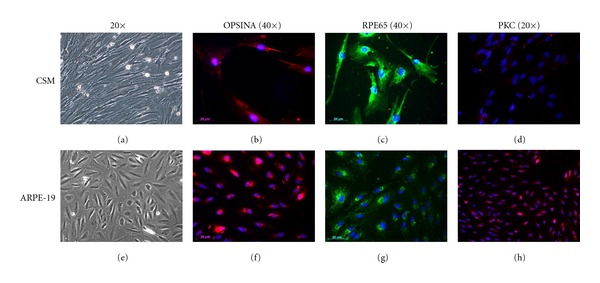
Immunofluorescence analysis of MSCs (a) for the expression of retinal markers Opsin (b), RPE65 (c), and PKC (d), after 14 days of culture, compared to ARPE 19 cells, used as controls (e, f, g, h). Original magnification 20× (a, d, e, h) and 40× (b, c, f, g).

MSCs cultured with ARPE-19 SN were evaluated by immunofluorescence analysis for the expression of retinal markers RPE65, Opsin, and PKC, after 7 and 14 days.  Figure 6 shows that, after 14 days, basal MSCs express retinal marker levels comparable to those of ARPE 19 cells, used as controls.

### 3.6. Drug Evaluation on MSCs in the Presence of Retina Cells

**Figure 7:**
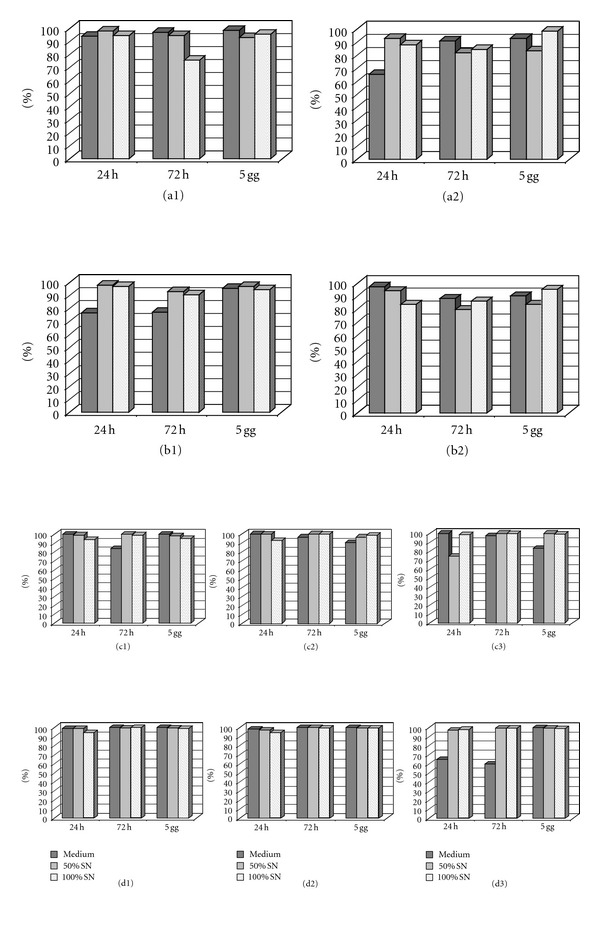
Viability conditions at different concentrations of drugs in the presence of ARPE-19 SN. (a1) TA 0.01 mg/mL; (a2) TA 0.1 mg/mL; (b1) AF-TA 0.01 mg/mL; (b2) AF-TA 0.1 mg/mL; (c1) IVT 0.01 mg/mL; (c2) IVT 0.1 mg/mL; (c3) IVT 1 mg/mL; (d1) DEX 0.01 mg/mL; (d2) DEX 0.1 mg/mL; (d3) DEX 1 mg/mL.

On the bases of the previous results we decided to test if in a “retinal like microenvironment” it would be possible to observe a protective effect of humoral substance on MSCs. To these purpose we tested the previous described culture condition of MSCs and steroid drugs, using conditioned medium obtained from the retinal cell line ARPE-19. We also tested different percentage of conditioned medium. In the presence of ARPE-19 SN the viability was better with all drugs. With TA, the effect of ARPE-19 SN was evident at 0.1 mg/mL after 24 hours, with AF-TA at 0.01 mg/mL after 24 and 72 hours. There was no effect on IVT and dexamethasone, but, at 1 mg/mL, the toxic effect after 24 and 72 hours was mitigated after retinic SN exposure (Figure 7). Where the effect of SN was evident, this was higher with 50% of SN than 100% of SN. 

**Figure 8:**
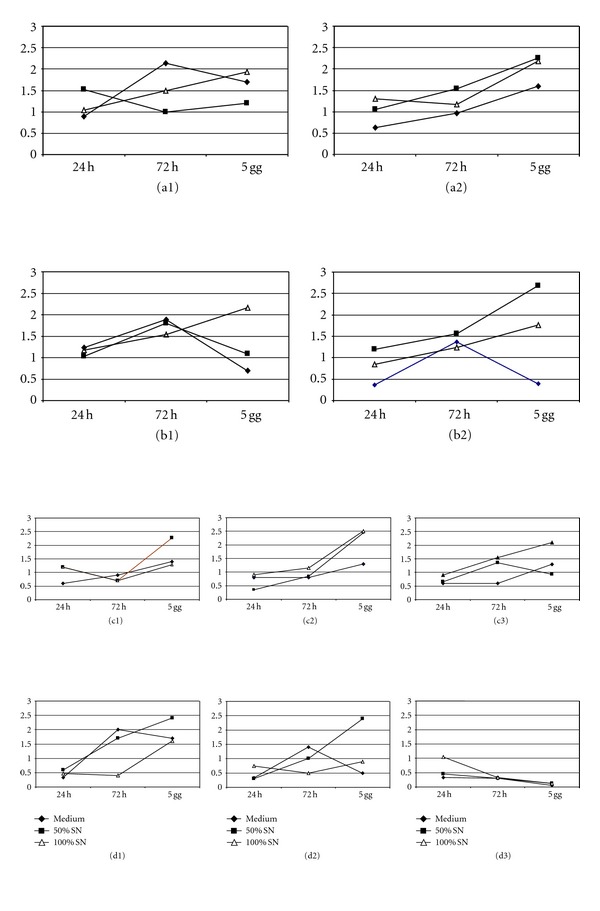
Growth rate at different concentrations of drugs in the presence of ARPE-19 SN. (a1) TA 0.01 mg/mL; (a2) TA 0.1 mg/mL; (b1) AF-TA 0.01 mg/mL; (b2) AF-TA 0.1 mg/mL; (c1) IVT 0.01 mg/mL; (c2) IVT 0.1 mg/mL; (c3) IVT 1 mg/mL; (d1) DEX 0.01 mg/mL; (d2) DEX 0.1 mg/mL; (d3) DEX 1 mg/mL.

As far as cellular growth is concerned, in the presence of TA, the most advantageous condition was at 0.1 mg/mL with 50% of SN. With AF-TA 0.01 mg/mL the effect of SN was positive in the presence of 100% SN, while at 0.1 mg/mL the best effect was with 50% SN. With IVT, the cellular growth was advantageous at 0,01 and 0,1 mg/mL in the presence of SN 50%, and at 1 mg/mL with 100% SN. The effect of 50% SN on cultures with Dexamethasone was positive at 0,01 and 0,1 mg/mL (Figure 8). The growth of MSCs in the presence of IVT or Dexamethasone seemed more correlated with the culture microenvironment (MSC medium versus ARPE-19 SN), with the presence of SN 50% resulting more advantageous. 

The expression of MSCs antigens did not change in the presence of ARPE-19 SN.

## 4. Discussion

The use of MSCs in regenerative medicine is a promising therapeutic approach for diseases characterized by a loss of retinal epithelium pigmented cells and photoreceptors, such as AMD. There are few treatment options for the dry form of AMD, while for the exudative form they are time consuming, expensive, and only of limited benefit to most patients. Therefore, the possible scope of a cell-based therapy is rather vast. AMD is a perfect candidate for the application of stem cell therapy in order to replace missing cells or to delay their degeneration. This study aimed at observing the behavior of MSCs in different culture media and in combination with corticosteroid drugs commonly used in clinical practice, to evaluate the toxicity, and then highlight the beneficial dose. Previous studies for other nonocular diseases tested the use of stem cells in combination with corticosteroids, pointing to positive effects on cell adhesion, proliferation, and viability.

These drugs may thus stimulate the proliferation and differentiation of MSC according to the complex environmental conditions [21]. However, very little is known about the initial events directed by corticosteroids that set the process in motion. Therefore, exploring the impact of these drugs on MSCs holds promise, for revealing important details of stem cell biology and for finding new fields of possible therapeutic applications.

In this work we evaluated the toxicity of Triamcinolone acetonide, with or without alcohol, micronized intravitreal triamcinolone, and dexamethasone at different concentrations on MSCs with or without the retinal cell culture supernatant ARPE-19, studying their morphology, viability, cellular growth, and immunophenotype.

The data demonstrated that MSCs cultured with corticosteroid drugs maintain their peculiar characteristics, despite viability being compromised. These data match those in Shaikh's study on ARPE-19 cells, reporting a correlation between TA concentration and cell loss [8], and those in Oh's study, which showed that even short periods of exposure to TA inhibited the proliferation of fibroblasts and RPE cells, resulting significantly toxic to confluent RPE cells [7].

A data evaluation of our study on morphology, viability, cell growth, and immunophenotype showed a different behavior of MSCs in the presence of the three drugs used, tested at the same concentrations and under the same culture conditions. The data showed a toxic effect of the drugs, mainly due to the higher concentration.

Comparing all the drugs, dexamethasone was the least toxic corticosteroid used in this study. Dexamethasone is a synthetic glucocorticoid frequently used in the treatment of severe inflammatory diseases with positive effects on the differentiation of mesenchymal progenitor cells into osteoblasts. In our study, dexamethasone at low concentrations was the least toxic drug, according to data from Song and Denis's study [24], showing that dexamethasone reduces or eliminates cell density-related apoptosis on MSCs. 

The microenvironment is also important. The use of the ARPE-19 retinal cell culture SN was an attempt to create a specific microenvironment in which to study the characteristics of MSCs and the effect of drugs compared with control cultures in MSC Medium. The presence of corticosteroid drugs and the lack of a culture medium typical of MSCs (100% SN), was first, a brake on cell growth, but also a factor that promotes differentiation. After 5 days, the most advantageous condition for MSC growth was culture with retinal SN 50%, irrespective of the drugs used. The most beneficial effect of 50% SN compared to 100% SN might be explained by considering that a lack of the standard culture medium for MSCs, might limit cell growth but promote differentiation in retinal direction, favored by the presence of a tissue-specific environment. Our study seems to confirm that a specific extra-cellular environment can protect MSCs from drug toxicity.

The presence of ARPE-19 cell line SN 50% and 100%, then the presence of trophic factors released by the retinal cells, seemed to help the cells to better preserve the typical morphology, and the precipitates were lower compared to standard culture media.

The use of immunofluorescence staining allowed us to identify the expression of specific markers expressed by retinal pigment epithelium cells (RPE65, opsin and PKC) on MSCs cultured with retinal SN, proving there is real potential for differentiation towards a retinal lineage when there is a suitable environment. Studies on the differentiation potential of MSCs are controversial. Although one study found that MSCs differentiated into cells resembling microglia rather than retinal neurons [17], other studies have shown that MSCs differentiate into retinal neurons *in vivo* and *in vitro* [18]. Moreover, animal studies have also demonstrated that the sub-retinal transplantation of MSCs delays retinal degeneration and preserves retinal function [19].

MSCs might be useful in cell therapy, particularly to slow down the loss of function through the production of neurotrophic factors and promote the survival of photoreceptors. MSCs seem to confirm a role in supporting cell expansion, cell reactivation of immunosuppression and neuroprotection. All these features might be supported by the concomitant use of specific drugs, such as corticosteroids.

The corticosteroid drugs tested in this study induce cell death only at high concentrations. Cell growth, viability and the functional properties of MSCs were good in the presence of low concentrations of drugs.

Recently, some reports demonstrated the clinical feasibility of the intravitreal administration of autologous bone-marrow-derived mononuclear cells in patients with advanced degenerative retinopathies [25, 26]. Siqueira conducted a prospective phase I trial to investigate the safety of intravitreal ABMC in patients with RP or cone-rod dystrophy, with promising results [27].

Our *in vitro* study demonstrated that high doses of corticosteroid drugs have a negative effect on MSCs. This effect was reduced on low pharmacological doses and in the presence of a conditioned media. Further studies are needed to improve our *in vitro* studies, and new drugs need to be tested, to understand the mechanism of interaction between MSCs and retina cells. Finally, for the purposes of a future clinical application, *in vivo* studies are necessary to study the potential role of MSCs for the treatment of AMD.

##  Conflict of Interests

Authors do not have any actual potential conflict of interest.
